# Inguinal canal angioleiomyoma: case report of a rare disease entity within inguinal canal

**DOI:** 10.1186/s13104-017-2800-9

**Published:** 2017-09-06

**Authors:** Jianwen Liu, Rockson Wei, Xuefei Yang, Xinping Shen, Jing Guan, Joe King Man Fan

**Affiliations:** 1 Department of Surgery, The University of Hong Kong-Shenzhen Hospital, Shenzhen, China; 2 Department of Surgery, The University of Hong Kong, Li Ka Shing Faculty of Medicine, Hong Kong, SAR, China; 3 Department of Diagnostic Radiology, The University of Hong Kong-Shenzhen Hospital, Shenzhen, China; 4 Department of Pathology, The University of Hong Kong-Shenzhen Hospital, Shenzhen, China; 5 Department of Surgery, The University of Hong Kong, Li Ka Shing Faculty of Medicine, Hong Kong, SAR, China

**Keywords:** Angioleiomyoma, Inguinal mass, Inguinal neoplasm, Inguinal exploration, Case report

## Abstract

**Background:**

Angioleiomyoma is an uncommon benign soft tissue tumor and originates from the vascular smooth muscle. It often causes pain and is rarely found in inguinal region. We present a rare case of inguinal canal angioleiomyoma of a female patient who suffered from right groin pain for 4 years and mimicking inguinal hernia clinically.

**Presentation of case:**

A 53-year-old Chinese female patient presented with 4-year history of right groin pain which was exacerbated by movement. Magnetic resonance imaging was performed in view of atypical presentation and absence of cough impulse. Inguinal canal was subsequently explored by open approach and the mass was found arising from the posterior wall of the inguinal canal and measured 5.2 cm × 3.8 cm. The posterior wall was repaired by Bassini approach after the mass was resected en-bloc. Inguinal pain was resolved and no hernia was found during follow-up. Pathology of the resected specimen confirmed angioleiomyoma with clear resection margins.

**Conclusion:**

This is the first report of a case of angioleiomyoma of the inguinal canal, which presents as a painful mass. Magnetic resonance imaging should be considered when presenting history and physical examination does not confirm with the diagnosis of inguinal hernia. After inguinal canal exploration, suture or mesh repair should be performed to prevent weakening of posterior wall leading to inguinal hernia.

## Background

Angioleiomyoma (ALM) is a rare variant of leiomyoma (LM) originating from smooth muscle cells and containing thick-walled vessels. It usually occurs in the subcutaneous tissue of the extremities [1]. The mass can be dermal or subcutaneous, or may occur in the superficial fascia of the extremities. Pain is documented as a primary concern in 60% of patients [2]. It commonly presents as painful mass and has never been described to be found in the inguinal region. We present a case of inguinal canal angioleiomyoma of a Chinese female patient who suffered from right groin pain for 4 years and mimicking inguinal hernia clinically. This is probably the first case report of such rare disease entity in the world.

## Case presentation

**Fig. 1:**
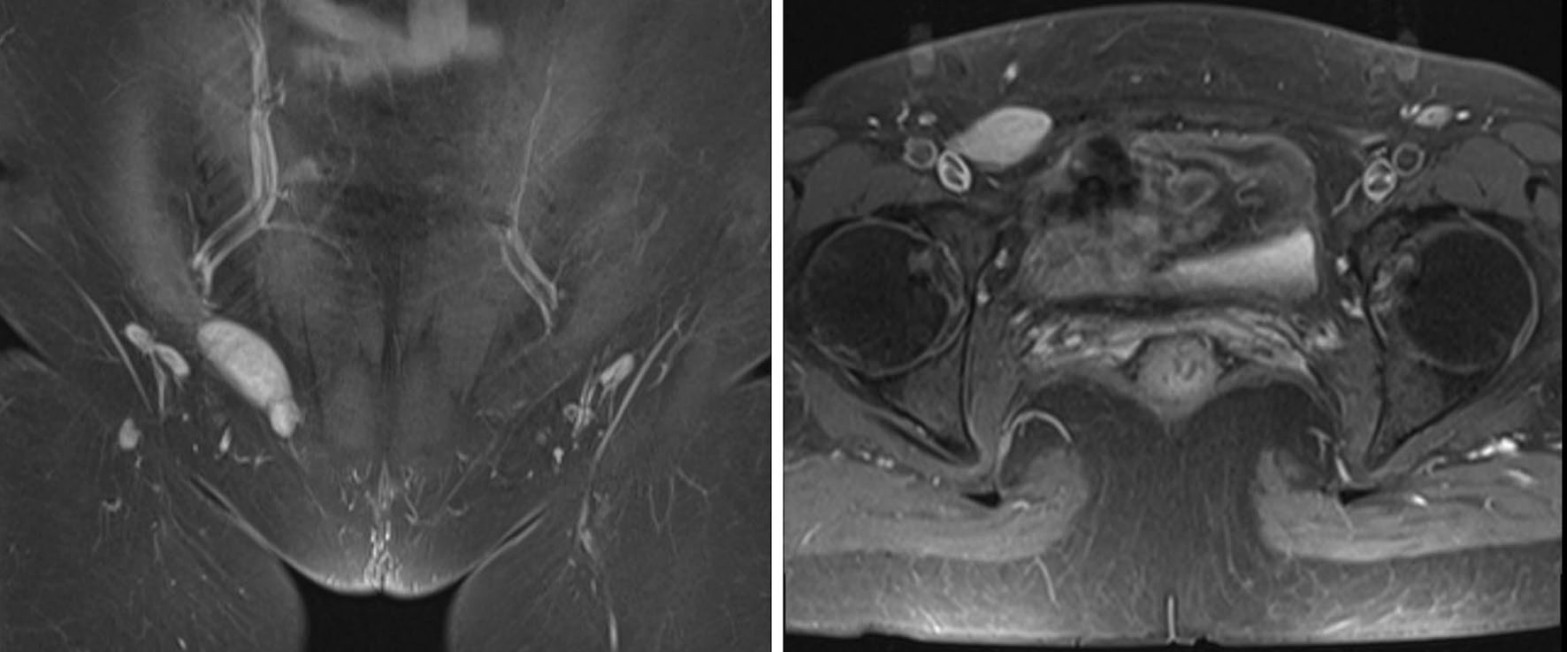
Enhanced magnetic resonance imaging showed that an intermediate enhanced mass located in right inguinal canal

A 53-year-old Chinese female patient with good past health presented with 4-year history of right groin pain which was exacerbated by walking and exercising. There were no other associated symptoms such as radiation of pain nor lower limb weakness, and there was no reported history of trauma or heavy weight lifting. She was referred to general surgical clinic for suspicion of inguinal hernia as a mass was felt over right groin by the patient. However, upon physical examination there was no cough impulse nor inguinal mass was found. In view of the atypical presentation of “inguinal hernia”, magnetic resonance imaging (MRI) was performed in August 2013. In view of suspicion of pathology originating from the round ligament, MRI would be ideal for soft tissue differentiation. A mass of 3.2 cm × 0.8 cm in size was found in right groin area, which was in the right inguinal canal and originated from the muscle of the posterior wall. It was isointense T1-weighted and hyperintense in T2-weighted images. The initial diagnosis was a “round ligament cyst” and she was initially managed conservatively with painkillers. In view of persistent pain, another set of MRI was performed 3 years later and it showed that the “cyst” was actually a solid mass with complete encapsulation. The size of the mass has increased from 3.2 cm × 0.8 cm to 5.2 cm × 3.8 cm over the 3 years. Differential diagnoses included round ligament cystic lesion, soft tissue tumors and hemangioma (Fig. 1).

**Fig. 2:**
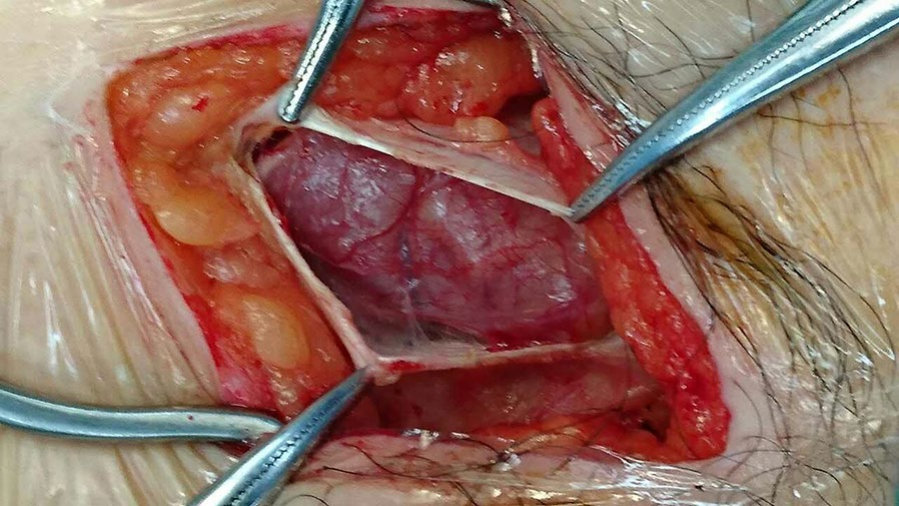
During right inguinal exploration, a mass with complete capsule was found below the external oblique fascia and originated from the posterior wall of inguinal canal

**Fig. 3:**
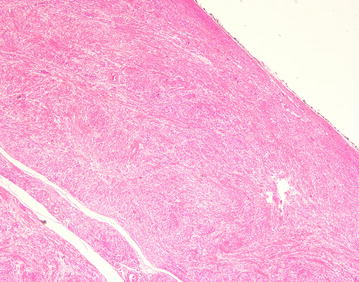
The tumor was composed of interlacing fascicle of spindle cells with interspersed abundant thick-walled blood vessels, H&E stain

**Fig. 4:**
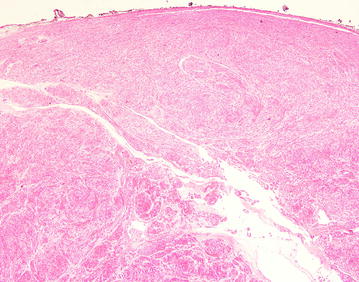
the tumor was composed of interlacing fascicle of spindle cells with interspersed abundant thick-walled blood vessels, H&E stain

**Fig. 5:**
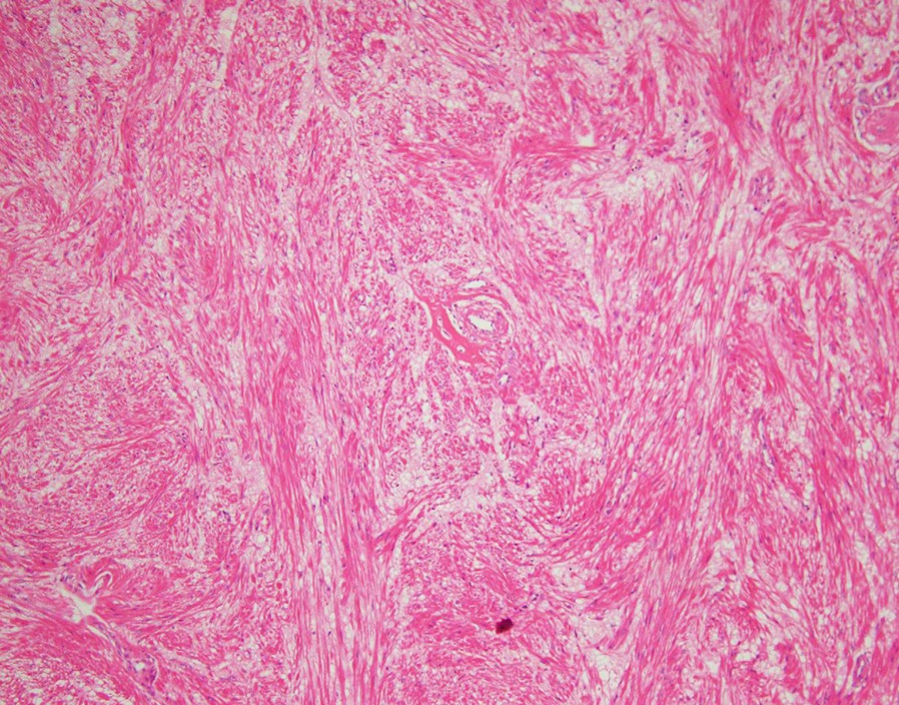
The tumor was composed of interlacing fascicle of spindle cells with interspersed abundant thick-walled blood vessels, H&E stain (high power field)

**Fig. 6:**
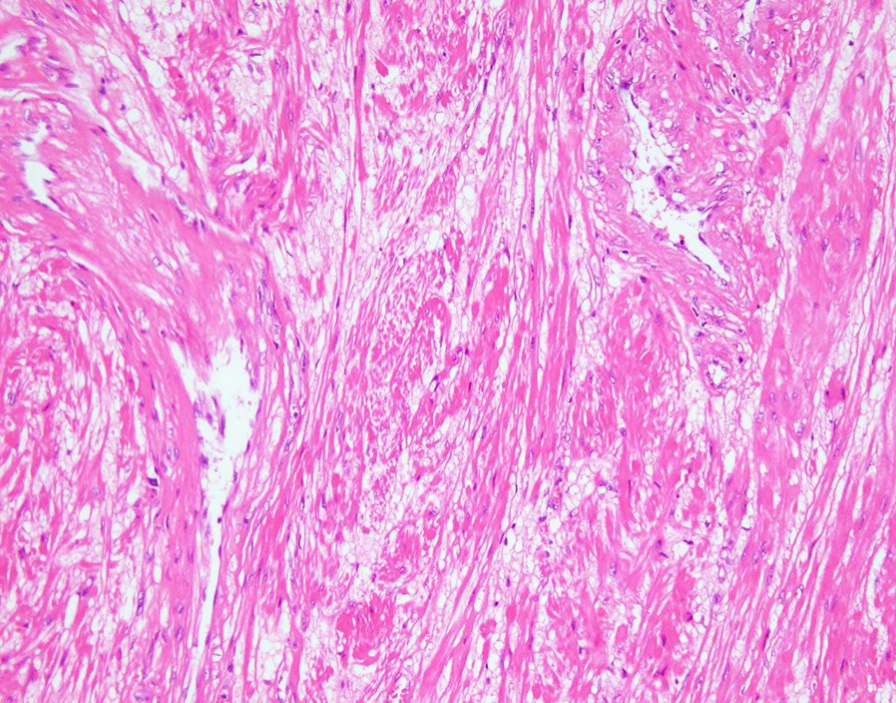
The tumor was composed of interlacing fascicle of spindle cells with interspersed abundant thick-walled blood vessels, H&E stain (high power field)

After discussion of pros and cons of surgical intervention, operation was arranged and inguinal canal was explored by open approach under general anesthesia. There was a 3.5 cm × 3 cm solid mass with intact capsule located in the right inguinal canal and originated from the muscle of posterior wall (Fig. 2). The mass was resected en-bloc. As there was weakening of posterior wall after resection Bassini repair was performed to prevent subsequent hernia formation. She was discharged on day 3 post operation with uneventful recovery. On histological examination, the tumor was composed of interlacing fascicle of spindle cells with interspersed abundant thick-walled blood vessels (Figs. 3, 4, 5, 6) and diagnosis of inguinal canal angioleiomyoma was made. During the follow up assessment at 3 months, groin pain was totally resolved and no recurrence of tumor nor occurrence of inguinal hernia was noted. In addition, ultrasonographical examination of the inguinal region showed no recurrence of the tumor. Currently our patient is on regular follow-up and surveillance.

## Discussion

Angioleiomyomas, also known as angiomyomas and vascular leiomyomas, are benign and classified by the World Health Organization (WHO) in 2013 as smooth muscle tumors. ALM account for about 4.4% of benign soft tissue tumors [3]. There are no studies indicating of malignant change potential in these tumors. This is the first case that ALM that is reported locating in inguinal canal. The most common presentation of ALM is a painful, solid, solitary subcutaneous nodule. It is mostly encountered in females in their second to sixth decade of life [4]. These lesions are slow growing and may take 10–15 years for patients to seek medical attention for the mass [5, 6]. 60% of ALM are found as a painful mass, which is the distinct clinical feature in this patient. The pain is thought to be related to local ischemia resulting from smooth muscle contraction [2]. The increasing in size of the swelling mass and pain exacerbated by walking and exercising, especially in the hand, are the most common complaint by patients [7]. Pre-operative diagnosis is difficult as there is an extensive list of possible differential diagnoses of similar lesions at this region. The use of ultrasound and MRI should be considered. In ALM, ultrasound examination shows well-defined margins and a homogenous structure, suggestive of benign nature of the lesion [8]. High resistance in intra-tumor arteries, which suggests the presence of muscular arteries, is the feature of angioleiomyomaon Color Doppler examination [9]. MRI scan was done in this patient rather than a computed tomography (CT) scan as it shows better resolution and soft tissue differentiation. In MRI ALM has a good soft tissue differentiation with distinctive features. T1-weighted MR images can show hypointensity areas and T2-weighted MR images showed mixed areas that were hyperintense and isointense to skeletal muscle. The hyperintense areas on T2-weighted MR images showed strong enhancement after intravenous contrast injection. Isointense areas on T2-weighted MR images did not show enhancement after intravenous contrast [10]. With superior image quality and soft tissue differentiation, MRI can give us a detailed road-map for surgical exploration. ALM causes minimal morbidity and local en-bloc excision is usually curative. Smooth muscle Actin stain in histological examination is helpful to portrait the smooth muscle bundles clearly [7].

This is the first reported angioleiomyoma in the inguinal canal. The differential diagnosis of similar cases in women included inguinal hernia, benign lesions such as fibroma, femoral artery aneurysm, hydrocele of canal of Nuck, leiomyoma, leiomyosarcoma, endometrioma, and other round ligament lesions [8, 11]. Malignant tumors such as leiomyosarcoma are also a possibility [12]. But most of the other causes usually do not present as inguinal pain, especially on exercising. Definitive diagnosis of inguinal masses is obtained through surgical excision. Complete excision usually curative but en-bloc resection with clear margins is mandatory in order to avoid recurrence of the tumor, which would complicates subsequent management. Neviaser reported a recurrence and malignant transformation 7 years after the initial operation requiring wide local excision [13]. Smooth muscle actin positivity can be used to rule out the tumors that do not originate from the smooth muscle in histopathological examination [14]. Cell surface protein markers CD31 and CD34, are helpful to differentiate ALM from other spindle cell tumor [15].

After our groin exploration, mesh repair was not done as the nature of mass was unknown during time of surgery. In case there is a tumor recurrence or need of further inguinal canal exploration, the presence of prosthetic mesh will render subsequent radical surgery more difficult. Moreover, we believed that a primary suture repair is sufficient to prevent hernia occurrence in this patient.

## Conclusion

Angioleiomyoma, which presents as a painful mass in approximately 60% of the cases, is rare in inguinal canal. MRI should be considered when presenting history and physical examination is not compatible with inguinal hernia or if pain is intractable and not response to conservative treatment. Simple excision is sufficient in managing the symptoms but after inguinal canal exploration, inguinal hernia may occur and suture or mesh repair should be performed.

## Abbreviations

ALM: angioleiomyoma
LM: leiomyoma
MRI: magnetic resonance imaging
WHO: World Health Organization
CT: computed tomography
